# Toxicological Risk Analysis in Data-Poor Countries: A Narrative Approach to Feed an “Awareness Raising—Community Empowerment” Vortex

**DOI:** 10.3390/medicina56110629

**Published:** 2020-11-20

**Authors:** Chiara Frazzoli

**Affiliations:** Department of Cardiovascular and Endocrine-Metabolic Diseases and Ageing, Istituto Superiore di Sanità, Via Giano della Bella 34, 00162 Rome, Italy; chiara.frazzoli@iss.it; Tel.: +39-4990-4133

**Keywords:** prevention, Africa, capacity development, environmental health, food safety, food security, toxicology

## Abstract

*Background and objectives*: With globalization of culture and products, choices and behaviors associated with the unawareness of toxicological risk factors result in human and environmental toxic exposures along with health disparities. Toxic exposures are risk factors for malnutrition and diseases, impairing the chances of being healthy and having a healthy adulthood for current and next generation(s). Increasing research funds, infrastructures, analytical data and risk assessment is a reality well worth attention in sub-Saharan Africa. These countries are still unprotected nowadays and are particularly exposed and data-poor in respect to risk factors (e.g., neurotoxicants, immunotoxicants and endocrine disruptors). This paper presents how—based on scientific literature—low-resource countries may achieve more with less. As one of the world’s most important emerging markets, Africa can, and should, assess the benefits and risks of modernity versus tradition and ask for safe and quality products at affordable prices while producing safe and nutritious foods. *Materials and Methods*: Exempla and experiences of risk analysis based on participant observation in field anthropological research, consumer safaris and reportages in the field of food safety, environmental health and consumer products are discussed in terms of “narrative prevention” and its power to highlight previously unrecognized/overlooked real-life risk scenarios. Knowledge return initiatives are discussed in light of their power to feed awareness raising, informed choice and empowerment of communities. *Results*: In some cases, data exist but remain too sparse, unknown or underused; in other cases, the information is totally neglected. When there is international scientific evidence, a diagnostic risk assessment is feasible. Despite significant resource constraints, properly science-driven targeted reportages in data-poor countries can bridge the gaps between international scientific knowledge and the implementation of relevant findings in an “awareness-empowerment vortex”. When a clear message promoting healthy choices and behaviors is given, African communities are ready to respond. *Conclusions*: Poor skills are an avoidable consequence of low national income. Narrative prevention does not replace scientific research but stimulates scientific research and toxicological risk analysis during the ongoing risk transition in Africa. While African populations increasingly aspire to improve life expectancy in health, increasing exposure to such new health risk factors in sub-Saharan Africa needs top-down choices for diseases prevention, One Health, as well as public awareness and empowerment towards everyday habits and health protective choices.

## 1. Introduction

Globalization of culture (lifestyle, diet, family, medicine and education) [1] along with globalization of consumer preferences and products (e.g., food, food-related products, packaging, cosmetics, clothing and materials) involves the globalization of toxicological health risk factors for human health, transgenerational health and environmental health [2,3,4]. The global burden of diseases indicates how the new era of co-morbidities (communicable and non-communicable diseases) is contemporarily evolving in risk factors. Choices and behaviors due to unawareness of toxicological risk factors result in toxic exposures contributing to health disparities [5]. This risk transition calls for attention to how toxic exposures affect the health of people via multiple and various pathways and aggregated effects.

In sub-Saharan Africa, limited coverage exists on topics such as toxicological risk analysis in terms of food safety, environmental health and consumer products. Evidence-based prevention, i.e., the process of making decisions based on the best available scientific evidence, encounters, in Africa, the problem of data poverty due to severe constraints in scientific research funding. Toxic exposure assessment is usually difficult and costly. In most cases, it is not necessary to repeat hazard characterization. Benchmarks that are protective in economically advanced countries should be lowered in living scenarios that are more stressed by deprivation and less protected by public health policies [5].

This paper proposes a feasible benefits and risks evaluation of products and practices introduced by modernity and globalization in African markets and environments. Leaving the introduction of modernity in the hands of non-African countries may not always be a healthy and manageable choice in the long run [5]. The leverage of informed choice is imperative at all levels (individuals, communities and policy makers) to compare the benefits of modernity with those of tradition and guide the transition in the globalized world. As emphasized by a talented African historian, Ki-Zerbo, the endogenic development of the continent requires the recovery of African cultural heritage (including materials, living environments, lifestyle and foods) against the globalization of culture [6]. It is worth noting that the tremendous positive implications offered to the African continent by its one billion consumers in need of, and in search of, all kinds of products will favor this attitude. As one of the world’s most important emerging markets, Africa can, and should, ask for safe and quality products at affordable prices [7].

## 2. Materials and Methods

As argued by Berrino, in science, “evidence” is the sum of scientific observations corroborating or rejecting a hypothesis, whereas in the Italian language, the term “evidenza” means “the quality of everything that you understand immediately without needing proof” [8]. The approach based on “evidenza” is applicable to feed science-based risk assessment in evidence (i.e., data)-poor scenarios [5], where the weakness of public health prevention policies makes people highly susceptible to toxicological risk factors. According to the general focus of observing the risk transition (i.e., exposure scenarios associated with modernization and tradition), participant observation in anthropological and ecological field research [9] followed two main thematic topics:

1. Kitchen toxicology.
2. Environmental toxicology.

Materials collected so far mainly consisted of:

(a) *Evidenza* collected during field surveys of consumer products recently banned in economically advanced countries.
(b) *Evidenza* of pollution of (food producing) the environment (e.g., photographic reportages near to electronic waste (e-waste) dumping or recycling sites).
(c) Check lists filled during inspections in farms, live animal markets, abattoirs and along food transport chains.
(d) Consumer safaris in market, collective and home cooking and street food.
(e) Photographs submitted by researchers and students of public and private universities and research institutions in sub-Saharan Africa during narrative prevention contests (theme “*food safety … from your sight*”).

Real-life risk scenarios of daily repeated exposure experienced by people were prioritized in the material selection. After focus group discussions (students, University of Yaoundé I, Cameroon; University of Port Harcourt, Nigeria) for the characterization of the identified issues, relevant scientific literature was critically reviewed, specific risk factors were described and discussed and feasible good practices or recommendations were itemized in peer-reviewed papers.

Awareness generated by *evidenza* was spontaneously followed by the purpose of developing knowledge return phases for the empowerment of communities. General criteria for identification of target audience were pivoted on (i) site-specific *evidenza* to allow people to recognize familiar exposure scenarios, (ii) progeny health as an asset to generate interest in preventive health seeking behaviors and (iii) community change as driver for individual effective behavioral change. Knowledge return activities based on *evidenza* on consumer products issues took place in school communities, whereas knowledge return activities based on the *evidenza* on chemicals in the environment took place in e-waste and farmers communities.

Such experiences of the science–society dialogue were characterized by (i) science-driven-context-situated narration of hazards and exposure risks, (ii) information for healthy behaviors and informed choice and (iii) good science-based practices in order to avoid or mitigate risk factors and protect (human and environmental) health.

### 2.1. Narrative Prevention: Limitations So Far

Based on the work done in collaboration with the non-governmental organization (NGO) Noodles (www.noodlesonlus.org) over the years 2010–2020, this paper conceptualizes the narrative prevention project, in which a feasible and low-cost science-based risk assessment allows an Africa-driven science–society dialogue towards the prevention of health risk factors in both production and consumption.

Some main limitations have been identified in the work done so far. These represent the objectives of work to be done in the near future:

(a) Skills in scientific photo reporting: quality and framing of pictures should properly document sources of contamination and possible pathways and context description.
(b) Standardized communication tools based on consensus guidelines and context-specific messages (e.g., fact sheets, illustrated cards and check lists) will allow quantitative comparison, reproducibility and monitoring of outputs in knowledge return initiatives in different target contexts and through different actors.
(c) Some degree of structure has been gained during the photo/video contexts promoted by the NGO (repository, rules, conditions and agreements). Systematicity should be given to all food chains from environment to consumption, based on both kitchen and environmental toxicology. Systematicity (i.e., planned and ordered procedure) in the collection and discussion of risk scenarios stimulating the interests of researchers will facilitate regular knowledge return activities. In turn, such systematicity and structure will allow for the identification of indicators and quantitative assessment of the outcomes and impacts.

### 2.2. Weaknesses and Strengths

**Figure 1:**
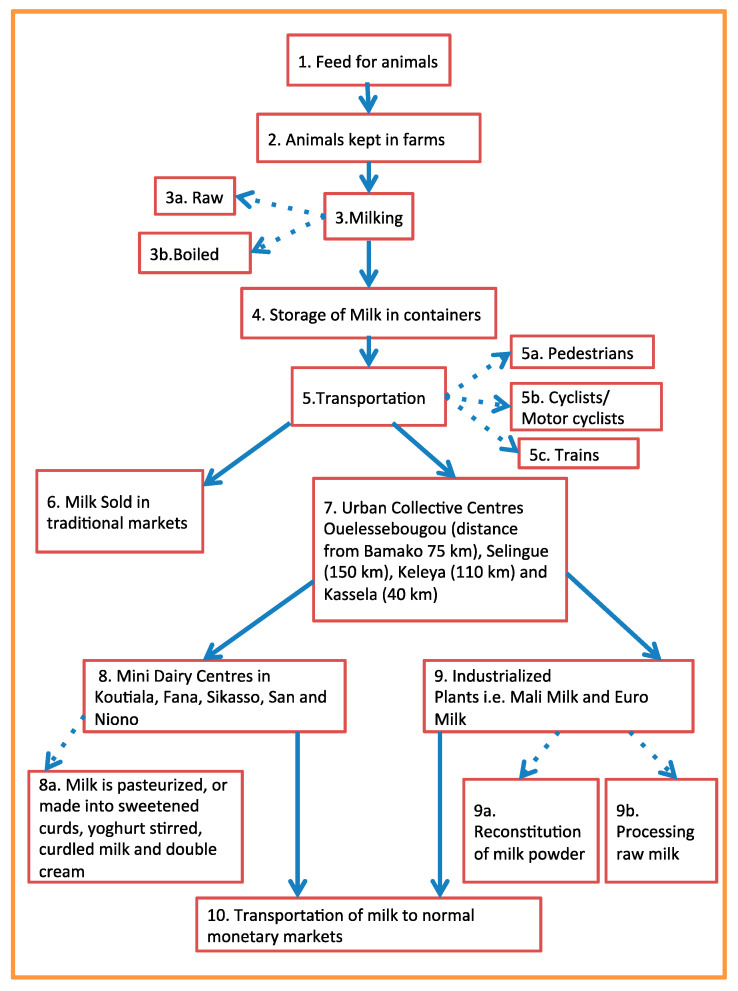
Flow chart depicting dairy chain in urban/peri-urban Malian areas (by Rachel Cheng, 2017).

While its weaknesses have not yet emerged, the approach has important strengths:

(i) It is low-cost; scientific literature (increasingly available in open access) and smartphones (increasingly available also in remote settings) make the identification and assessment of risk factors easy to handle with limited resources.
(ii) The systematicity exploits the widely known hazard analysis and critical control points (HACCP) approach. Flow charts depicting food chains from environment to consumption (e.g., Figure 1) can help with organizing the collection of *evidenza* and the identification of appropriate knowledge return initiatives. A flow chart assists to determine all the points of particular attention from environment to consumption along the specific food chain in question. At each point and passage, potential contaminations can be noted by investigating the actual surroundings.
(iii) The continuity of the approach from environment to kitchen clarifies how the prevention of human diseases passes for the care of the environment, including the health of food-producing animals, thus boosting the One Health perspective [10].
(iv) The approach exploits narrations that, in sub-Saharan Africa, remain the appropriate mode of transmission of values and knowledge to communities and families. Narratives connecting events or experiences have become a common health communication tool in recent years, and reportages can help audiences to understand health messages and find motivation to make behavioral changes [11].
(v) The methodology (awareness raising—community empowerment) was named “vortex” to recall the potential of continuing collective change, based on science and acted on by local researchers and citizens. The theory of the “healthcare vortex” has already shown how locally developed and delivered, locally adaptive, people-centered disease prevention systems may deliver highly effective and sustainable effects despite significant resource constraints [12].

### 2.3. African Perspective

Finally, this work should be intended as a proof of concept. It is noteworthy that:

(a) The work done so far is not meant to be exhaustive and it could not involve all scenarios and their prioritization. The enduring application of the narrative prevention approach will highlight new and unexpected exposure risk scenarios. Even if some general common features are shared by communities in sub-Saharan Africa, a prioritization of benefit-to-risk scenarios is feasible from a context-situated perspective. Traditionally-rooted behaviors, modernization rate, characteristics of dumping phenomena, target subgroups and exposure scenarios all change according to context-specific features (see, e.g., [9]).
(b) The approach is not intended to replace scientific data or efforts to strengthen the science of risk assessment in sub-Sahara Africa. It boosts risk analysis (i.e., risk identification, assessment, management and communication) and investments in research and analytical infrastructures, surveillance systems (including food safety, environment and consumer products) and Africa-led science.

## 3. Results

### 3.1. Awareness Raising

#### 3.1.1. Kitchen Toxicology

Nutrition transition from traditional diets (high in cereal and fiber) to more Western-like diets (high in energy, sugars, fat and animal-source food) entails the transition to new risk factors, such as toxic exposures to environmental and process contaminants [4]. The demand for foodstuffs with a given taste, texture or color may imply exposure to additives (e.g., flavoring or coloring agents), artificial aromas and preservatives and the risks of masked, poor quality.

Toxicant exposures may worsen the micronutrient status, especially during the womb-to-childhood development, by impairing organism programming and increasing the risk for health disorders in adulthood. Nutrition security is assured by a balanced diet, which is composed by nutritious foods that are free from environmental and process contaminants [4].

##### Traditional Nutraceuticals

**Figure 2:**
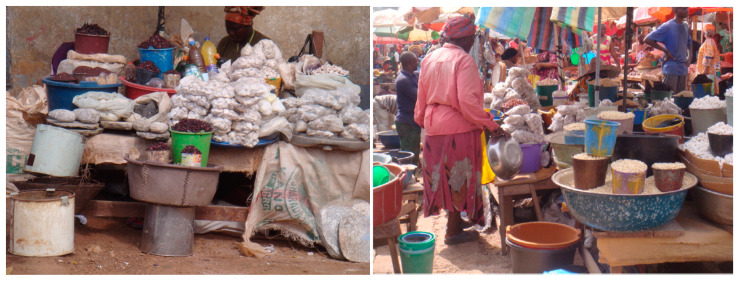
Geophagic soils vended in food markets in Cameroon, 2016 (by Guy Pouokam). “Narrative prevention project” (www.noodlesonlus.org) © courtesy of Noodles.

The relevance of environmental toxicology in nutrition is self-evident when geophagic communities are concerned. Market reportages interestingly proved the vending of geophagic clays, i.e., a sort of traditional nutraceutical [13], amongst food items in food markets (Figure 2). Further to risks due to the changed environment [14] (no more “organic” productions as naturally found in the near past), the practice of vending geophagic products among food items should be prohibited. Geophagic clays are seen as food or good substitute of food, especially (but not only) for pregnant women. In some cases, such vended soils present favorable health claims, i.e., statements about relationships between their consumption and favorable health effects. In reality, health claims must be prohibited in the absence of robust and relevant scientific evidence as well as prohibition of the sale of the geophagic clays. Consumers should be warned about potential (biological, chemical and physical) hazards of consuming soil. Possible dangers of mineral supplementation in the absence of ascertained deficiency should also be made known to the consumers [13]. Based on photo-captured information, safe and controlled (i.e., not geophagic and not self-given) dietary supplementation in individuals at risk of deficiency emerged as a health issue, as well as the need for prohibiting health claims on geophagic clays and their vending in food markets. A nutritious diet should not be replaced by nutraceuticals. When decisions have been taken on a nutraceutical/dietary supplement, appropriate usage should be monitored on the field. In the case of geophagic products, work is still ongoing towards a consensus strategy for their regulation. As described in the following and in “3.1.2. Environmental Toxicology”, the knowledge of the area/site of origin in question is crucial for the selection of geophagic soil or food ingredients for purchase.

##### Diet of Infants

To date, a known exemplum of an International Code of Marketing is the one on breast milk substitutes. Indeed, compliance should be monitored. Restrictions on breast milk substitute marketing, such as infant formulas, should be strictly monitored. Mothers should not be discouraged from breastfeeding and substitutes should be used safely only when it is needed. Breastfeeding avoids the risk of contaminated water to reconstitute baby formulas. Infant formulas should be monitored for their safety [15].

##### Food Contact Materials

In urban areas, mothers are increasingly using baby food products and baby bottles; however, awareness of the health risks due to exposure to substances leached from materials in contact with food is missing. It is surprising that even shopkeepers and healthcare personnel are unaware of the health risks of harmful baby bottles, like the mothers.

**Figure 3:**
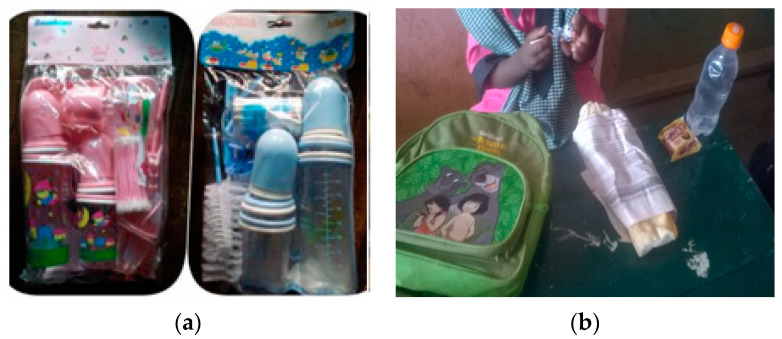
(**a**) Baby bottles sold in pharmacies, shops and markets, Yaoundé, Cameroon, 2014 (by Guy Pouokam); (**b**) School children with sandwiches wrapped in newspapers, Yaoundé, Cameroon, 2015 (by Philomina Fankam). “Narrative prevention project” (www.noodlesonlus.org) © courtesy of Noodles.

Bisphenol A (BPA) is a widely used industrial plasticizer with endocrine, mainly estrogenic, activities. BPA is present in the packaging of many baby food products (epoxy resins of glass bottle tops and lids, linings of metal cans holding powdered and ready-to-consume liquid baby formula) and in polycarbonate plastic baby bottles. BPA toxicology at low doses has been controversial. It is plausible that exposure to BPA in developing countries, which have sometimes aggressive conditions of use and more uncontrolled sources of exposure, is greater than in high income countries. As already proven by the mentioned baby bottles survey (both pharmacies and street vendors sell polycarbonate baby bottles, and pharmacists sell BPA and BPA-free baby bottles at the same price), a field survey corroborated how exposure is often more a matter of awareness than affordability [16]. Images captured another unsafe daily habit: wrapping sandwiches in newspapers (Figure 3b); it is known how newspapers release toxic substances (e.g., BPA) from the printing inks.

Products considered as hazardous in high-income countries should not be dumped in unprotected countries to dispose of stocks [5]. Reportages on consumer products in markets (Figure 3a) motivated an observational survey. In Cameroon, the survey raised ministerial attention when it demonstrated that plastic baby bottles with high availability, accessibility and affordability in all vending points (pharmacies, shops and markets) contain BPA in different regions of Cameroon and Nigeria [16]. The survey highlighted how baby bottles banned in many economically advanced countries worldwide for their possible adverse health effect on infants are dumped in unaware countries, including countries in sub-Saharan Africa. Such reportage brought out the need to define and spread good practices aimed at protecting bottle-fed children from exposure to BPA in polycarbonate bottles.

##### Toxic and Unsuitable Cookware and Utensils

**Figure 4:**
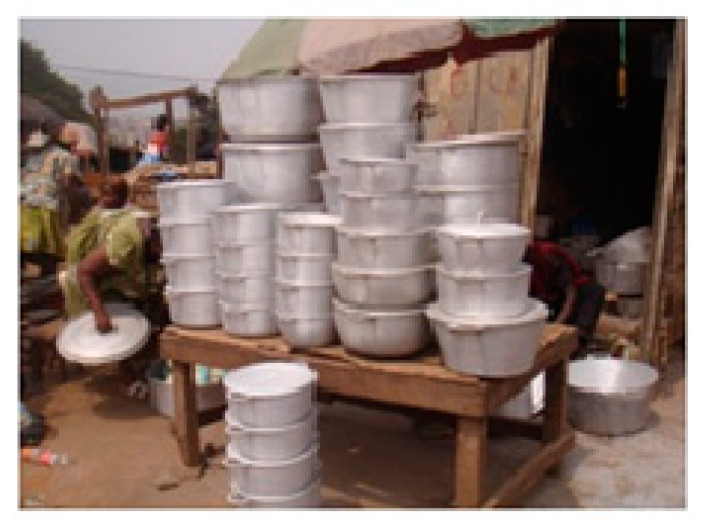
Aluminum pots and pans commonly on sale at the Mokolo market, Yaoundé, Cameroon, 2016 (by Guy Pouokam). “Narrative prevention project” (www.noodlesonlus.org) © courtesy of Noodles.

**Figure 5:**
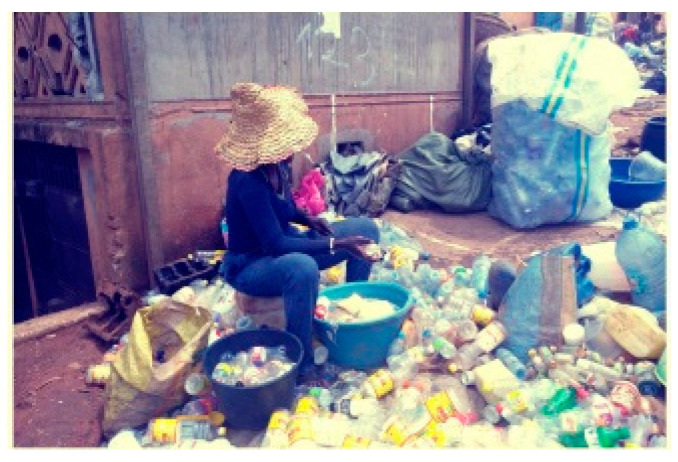
Washing of plastic bottles for reuse, Cameroon, 2017 (by Kateu Tsegouang Franck Arnold). “Narrative prevention project” (www.noodlesonlus.org) © courtesy of Noodles.

The issue of vending and use of unprotected aluminum or copper pots and pans (often from informal recycling markets, e.g., derelict cars) for cooking emerged during market reportages as a widespread source of toxic exposure in sub-Saharan Africa (Figure 4). Disposable plates made up of plastics, such as polythene, polypropylene, polystyrene, polycarbonate and polyvinyl chloride, pose health risks due to the release of toxic chemicals, such as BPA, melamine, vinyl chloride and phthalates. The usage of disposable plastics also imposes microplastic pollution on top of the harmful food contact effects on humans. A huge amount and variety of materials, mostly plastics, is in contact with food while it is prepared, stored, transported and sold. For instance, plastic bottles and bags collected from waste are washed and reused to fill up with drinking water and local juices (Figure 5). Containers that have contained pesticides or other toxic materials are reused to carry food or water in areas where access to storage containers is limited. Food contact materials (e.g., resins coating metal cans, cookware, plastic containers improperly used in microwave ovens, utensils, glasses and food packaging materials) should only be those approved for use in food facilities, purchased from formal retailers and in good, undamaged and not in a deteriorated state. Some options for self-reliance in the informal sector have been started in Africa to face plastic pollution [17]; when the risks of adverse and unintended health effects are addressed, the transition to a circular economy provides a major opportunity to yield substantial health benefits, including the reduction in negative environmental impacts [18].

##### Rethink and Reuse of Traditional Organic Materials

**Figure 6:**
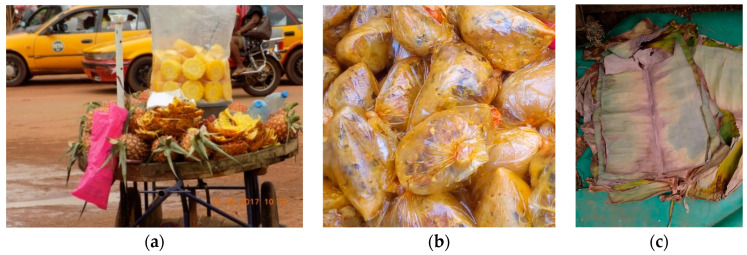
(**a**) Ambulant vending of sliced pineapples in waste plastic bags, Cameroon, 2017 (by Sicheu Fany Alliant Chany); (**b**) Moi Moi, in polythene, Abakaliki, Nigeria, 2020 (by Chidi Eze Eze); (**c**) Banana leaves as traditional food packaging, Cameroon, 2020 (by Aristide Kamda). “Narrative prevention project” (www.noodlesonlus.org) © courtesy of Noodles.

Plastics are seen as a sign of modernity, whereas (often) safe materials coming from tradition are intended for poor people in the villages (e.g., banana leaves) (Figure 6a–c). Critical evaluation of products locally made with waste materials has helped local researchers to rethink about the value of natural, local resources, e.g., making disposable plates out of plant leaves that are renewable, biodegradable and rich in antioxidants and medicinal values [19]. Stainless steel, bamboo and cotton often provide toxic-free and eco-friendly solutions to plastics.

##### “Food Safety … from Your Sight” Photo Contests

**Figure 7:**
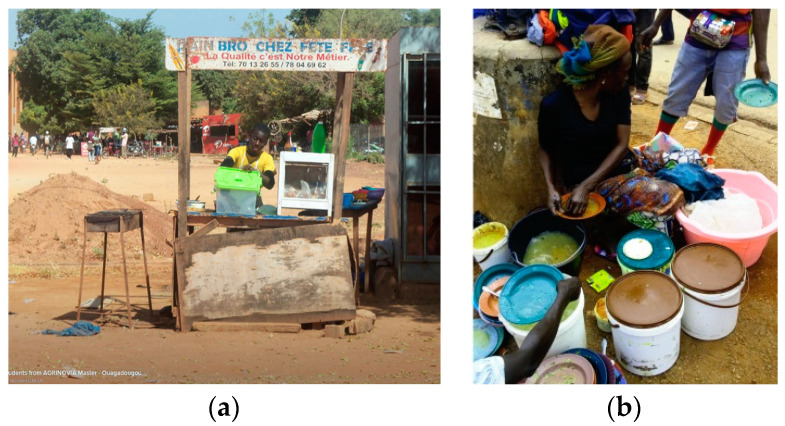
(**a**) Street food in Ouagadougou, Burkina Faso, 2012 (by Ilaria Proietti); (**b**) Couscous, vegetables, rice and groundnuts soup in Yaoundé, Cameroon, 2016 (by Kateu Tsegouang Franck Arnold). “Narrative prevention project” (www.noodlesonlus.org) © courtesy of Noodles.

**Figure 8:**
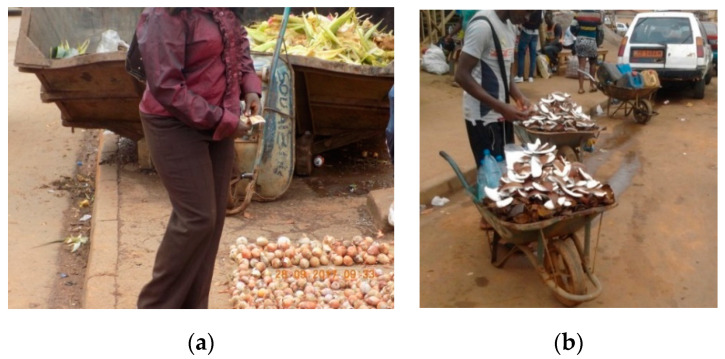
(**a**) Onions exposed on the street close to a waste discharge/dumpsite, Cameroon, 2017 (by Sicheu Fany Alliant Chany); (**b**) Coconut pieces sold on trafficked street, Cameroon, 2017 (by Isabelle Bouelet Ntsama). “Narrative prevention project” (www.noodlesonlus.org) © courtesy of Noodles.

**Figure 9:**
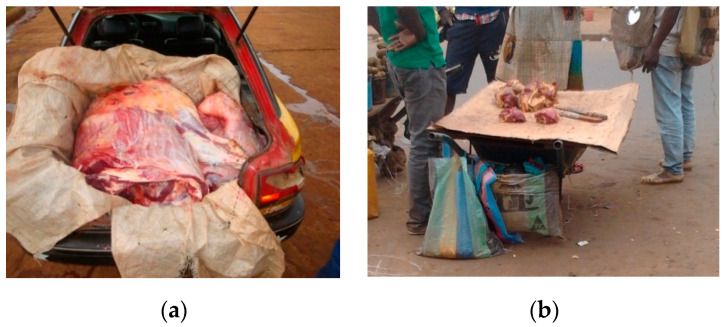
(**a**) Fresh beef wrapped in dirty plastic and transported from the abattoir to markets in a taxi, Cameroon, 2018 (by Tondji Mbadjitat Christelle); (**b**) Fresh beef for sale on cardboard on a wheelbarrow in the street, Cameroon, 2018 (by Isabelle Bouelet Ntsama). “Narrative prevention project” (www.noodlesonlus.org) © courtesy of Noodles.

Interestingly, subjects chosen by local scientists and university students in the photos/videos contests “*food safety … from your sight*” organized by the NGO Noodles (2017–2018) proved local concerns on the following:

- Ready-to-eat foods sold in streets, public places and markets (Figure 7a,b).
- Inadequate means of food transportation (e.g., wheelbarrows and common taxis; Figure 8a,b).
- Vending of raw foods (meat, fresh and dry fish and dairy products) in crowded and polluted environments and close to dumpsites (Figure 9a,b). In the poorest communities in sub-Saharan Africa, 80–90% of waste is not collected for safe disposal. Waste usually ends up in illegal dumps on streets, open spaces and wastelands, often subjected to burning and production of dioxins [20].

##### Good Cooking Practices

**Figure 10:**
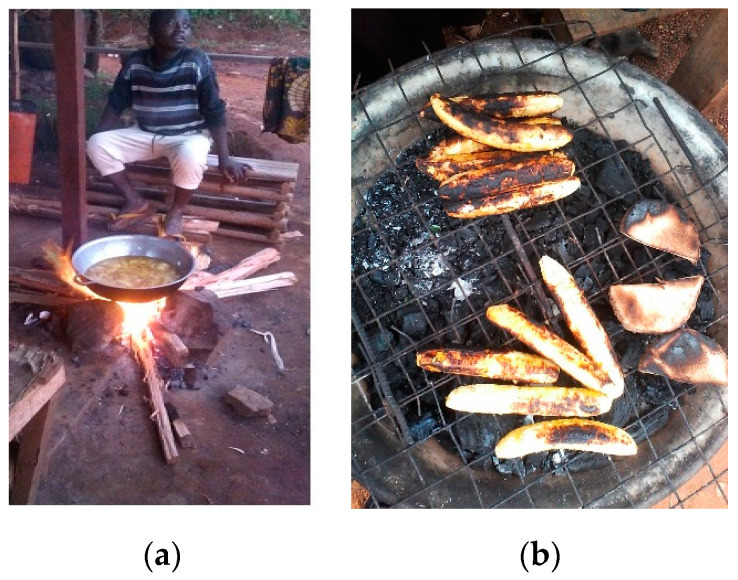
(**a**) Doughnuts cooked on an open-air wood fire in an aluminum pot with reused refined oil, Cameroon, 2017 (by Saha Foudjo Brice Ulrich); (**b**) Roasted plantain or Bole, a street food in Port Harcourt, Nigeria, 2017 (by Igbiri Sorbari). “Narrative prevention project” (www.noodlesonlus.org) © courtesy of Noodles.

**Figure 11:**
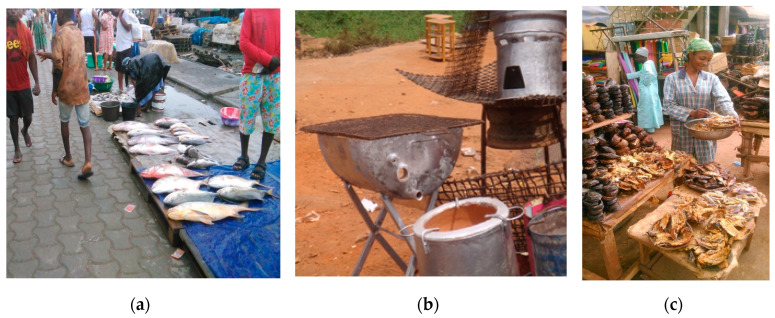
(**a**) Fresh fish sold at the side of the street, Cameroon, 2017 (by Kouandou Mohamed); (**b**) Traditional dryer, Yaoundé, Cameroon, 2016 (by Guy Bertrand Pouokam); (**c**) Dry fish bought and resold in pieces or heap Cameroon, 2017 (by Kouandou Mohamed). “Narrative prevention project” (www.noodlesonlus.org) © courtesy of Noodles.

In kitchen toxicology [21], not only materials in contact with food are considered but also environmental and process contaminants. Good practices applied to food cooking (boiling, stir-frying, steaming, roasting, grilling, frying, baking, broiling, barbecuing, microwaving, smoking or sun drying) can prevent repeated and daily toxic exposure and anti-nutritional factors and, hence, maximize the intake of nutritional value in food [21]. Further to chemical leaching from food contact materials, recipes (from purchase of raw ingredients through to food handling and cooking) can prevent contaminations from environmental and process toxicants; appropriate storage can prevent toxins (e.g., mycotoxins and histamine in scombroid fish) [21,22]. For instance, high-temperature cooking or overcooking high carbohydrate and protein-rich foods may generate toxic process contaminants (e.g., carcinogenic polycyclic aromatic hydrocarbons (PAHs), acrylamide, benzo(a)pyrene and heterocyclic aromatic amines; Figure 10a,b) [23]. With regards to the fish cycle (e.g., Figure 11a–c), for instance, partial cooking in the stove prior to smoking could reduce smoking time and risks generated by bad practices [21]. Street food presents a specific point of attention [22].

##### Kitchen Environment and Surroundings

**Figure 12:**
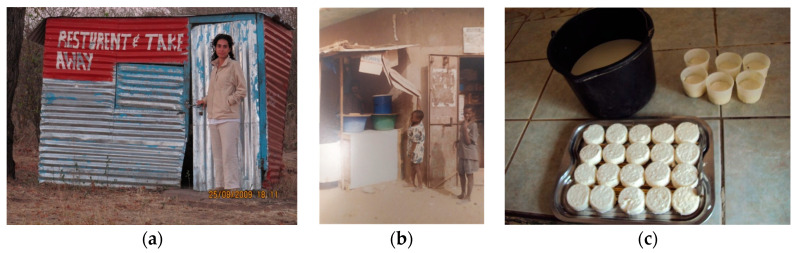
(**a**) Takeaway restaurant, Botswana, 2009; (**b**) Milk vending, Dakar, Senegal, 2000 (by Chiara Frazzoli); (**c**) Small dairy in Ouagadougou, Burkina Faso, 2012 (by Ilaria Proietti). “Narrative prevention project” (www.noodlesonlus.org) © courtesy of Noodles.

The kitchen environment is crucial to protect food from polluted air (Figure 12a). Indoor space protects the kitchen from airborne chemicals, but indoor cooking over, e.g., charcoal or dried dung can result in substantial inhalation of particulate matter if the kitchen is not adequately ventilated. Food should be covered with clean material to avoid exposure to airborne chemicals (e.g., heavy metals, dioxins, PAHs and particulates) in dusts and automotive exhaust or waste burning fumes [21,22] (Figure 12b). Concerning surfaces, foods, dishes and utensils should be removed prior to fumigation and sanitization and residues of fumigants and sanitizers should be removed (Figure 12c). Appropriate storage conditions (temperature, time and moisture) can avoid, e.g., the growth of mycotoxin-producing fungi in cereals, dry fruits, spices, coffee and cocoa. Vegetables should be washed extensively to remove insecticides and pesticides. An excessive or wrong use of soap, disinfectants, biocides, insecticides or rodenticides may contaminate food.

##### Raw Foods at the Interface with the Environment

Based on local species, aquaculture farmers could rear safer local fish species; factors such as fish age, species and size may significantly change the benefits to risks ratio [24], whereas fishing and hunting should be limited to unpolluted areas. Adipose tissues and bioaccumulating organs (e.g., liver) could be removed by peeling fish, removing entrails and cutting away fat to avoid lipophilic contaminants [21].

##### Benefits of Food Safety Training

The easy procedure to open food businesses such as ‘quick service restaurants’, e.g., in Nigeria, motivated the explosion of numbers of fast food retail outlets (from mama puts and roadside cafes to suya sellers); however, the owners often operate without monitored standard practices to ensure safety and hygiene. Interviews highlighted how, analogously to operators and workers in restaurants, the work of street food vendors would benefit from training manuals for food handling, cooking and serving to ensure safety from biological, physical and chemical hazards and exploit the value of diversified and widespread offer of meals [22]. In Asian countries (e.g., Vietnam and Thailand), meals—street food included—generally follow the dietary pyramid and combine raw and cooked foods: here, some hygiene practices are already adopted and the price of street-cookery is lower than in African countries; this means that the market has found its route. People can spend time around a table with friends by eating good and safe foods. If properly analyzed and managed, risk factors in street-cookery in sub-Saharan Africa could be avoided through focused education as demonstrated to be successful in Asia [25].

#### 3.1.2. Environmental Toxicology

Interestingly, ready-made foods seem to be a great concern for local scientists and students. Limited attention is paid to the contamination of food-producing environments. Evidence of unhygienic and toxic environments is found at almost all corners where people produce and handle food. Only a few people pay real attention on what they see; the issue appears as common and near to normal.

##### Animal Keeping

**Figure 13:**
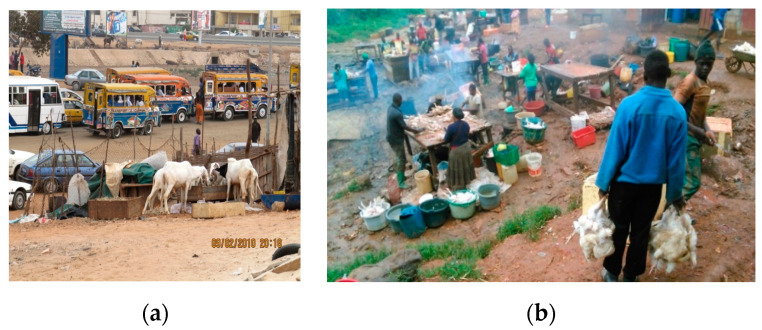
(**a**) Grazing of food-producing animals along the main route of vehicular traffic, Dakar, Senegal, 2010 (by Chiara Frazzoli); (**b**) Open market of live animals, Cameroon, 2018 (by Kouandou Mohamed). “Narrative prevention project” (www.noodlesonlus.org) © courtesy of Noodles.

**Figure 14:**
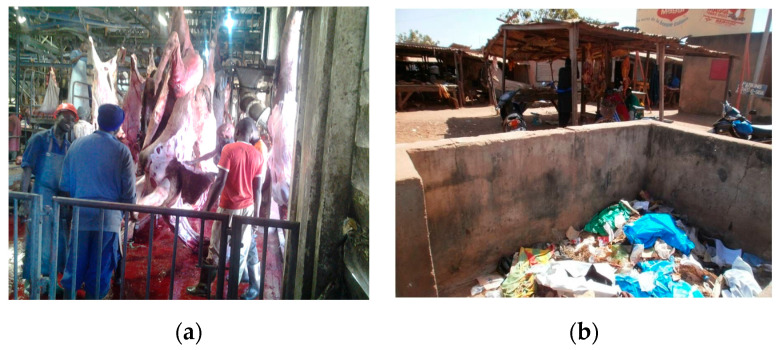
(**a**) Clean area of a slaughterhouse, N’Djamena, Chad, 2015 (by Kimassoum Djimadoum); (**b**) Open waste in proximity of a slaughterhouse, N’Djamena, Chad, 2015 (by Kimassoum Djimadoum). “Narrative prevention project” (www.noodlesonlus.org) © courtesy of Noodles.

**Figure 15:**
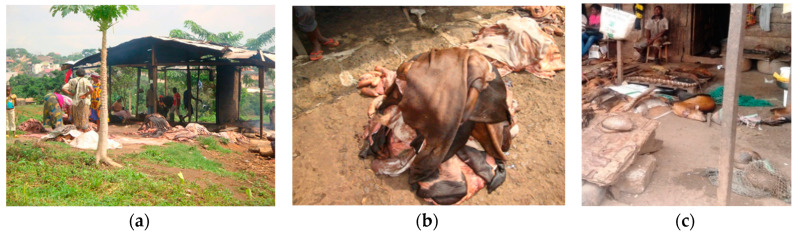
(**a**) Beef-processing hangar near the abattoir, Yaoundé, Cameroon, 2017 (by Christelle Tondji Mbadjitat); (**b**) Bovine skin ready for drying and ovals disposals at the abattoir, Yaoundé, Cameroon, 2017 (by Christelle Tondji Mbadjitat); (**c**) Bush meat bought from hunters and laying down before drying, Yaoundé, Cameroon, 2017 (by Isabelle Bouelet Ntsama). “Narrative prevention project” (www.noodlesonlus.org) © courtesy of Noodles.

The environment where animals are kept is crucial to avoid udder infection and uptake of environmental pollutants from watering and grazing, etc. [26,27]. Some seminal analysis of safety in food production [28] shows how, starting from the agro-farming scenario and its environment, important contamination (e.g., of cereals, fish, sea products, eggs, honey, shrimps, chicken, and feed ingredients) by toxic metals, mycotoxins, veterinary drugs’ residues or pesticides has been found, with both a direct (on health) and an indirect (on trade, economies, and livelihoods) impact. The role of chemical pesticides to support the life of agricultural communities is undoubted, but it is also true that there are risks of their excessive and unaware use. Feed contaminants are a broad issue, ranging from undesirable or unauthorized feed components or additives through to environmental pollutants and contaminants related to specific steps of production, such as storage or cross-contamination [29,30,31]. In the cycle of meat production, from pastures and farms, markets of live animals, stables and abattoirs, preliminary experiences (inspection-like; Figure 13, Figure 14 and Figure 15) in Cameroon and Chad reported interests by primary food producers towards hygiene and agro-zootechnical good practices as well as rudimentary systems for accounting for chemicals (type, quantities, points of sale origin and time of use) and checklists (environment; agricultural and zootechnical practices; animal health and welfare; food storage; waste management). Anthropic pollution of food chains by, e.g., vehicular traffic is widespread. Cutting of hides, drying of meat, abattoir garbage utilized as fertilizer and meat transportation are exempla of points of particular concern. For instance, hide is a major non-food product of animal origin with possible important public health problems (e.g., anthrax). Photographic reportages can facilitate the understanding of the history and status of food along its chain from farm to consumption (Figure 1) and the awareness of possible synergistic and cumulative contaminations.

##### Natural Toxins

**Figure 16:**
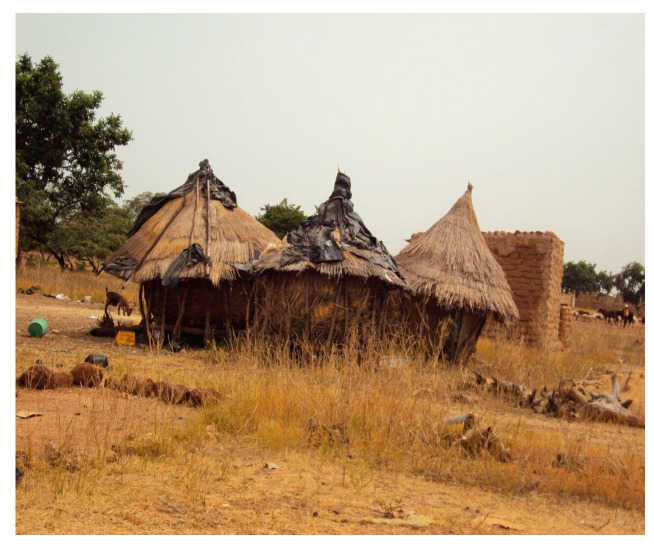
Granaries in Ouagadougou, Burkina Faso, 2012 (by Ilaria Proietti). “Narrative prevention project” (www.noodlesonlus.org) © courtesy of Noodles.

Good practices should be spread to handle the risk of natural toxins (e.g., Aflatoxins) [32,33]. It is known how Aflatoxin B1 (AFB1) contaminates feeds and constitutes a risk for the health of several farm animals, including fish. Besides correct feed storage to prevent mycotoxins (Figure 16), other good practices in the farm await implementation, e.g., proper use of biocides and preserving agents. Clean livestock feed holds the key to clean milk. Photos captured and clarified safe and unsafe conditions of use and techniques for reduction in AF contamination and prevention of AF formation during storage. For instance, silages for preserving green fodder are unsafe if anaerobic conditions are not strictly controlled (e.g., artificial drying in the wet seasons); transportation of grains in wet and closely packed conditions (lack of aeration) is unsafe [33].

##### Environmental Pollutions’ Impact on Food Chains

Chemicals are introduced (voluntarily or not) in the environment by agro-farming or anthropic activities, including disposal/recycling polluting practices. When a contaminated environment is involved, agricultural features are of paramount importance, such as pastures, feeds, main farm animal species and products (e.g., milk, meat and eggs), as well as size, distribution and type of farming. Whenever possible, grazing livestock should be moved to pastures and watering sources at a safe distance from contamination sources. Toxic contaminants in soil, air, and water carry over in aquatic lives, vegetables and crops and in food-producing animals at potentially harmful concentrations. Due to its possible magnitude, persistence and wide effects on communities, the direct manmade contamination of environmental compartments deserves due attention. For instance, breastfed infants’ toxic exposure to e-waste areas is severely higher than the WHO guideline values for carcinogenic and non-carcinogenic risks [34]. In these areas, the risk-to-benefit assessment of breastfeeding (otherwise a highly recommended practice) should be performed and alternatives (e.g., milk of nurses or milk from local farm animals) is advisable when the risks exceed the benefits. E-waste is a telling example of the complex pollution of food chains, especially for vulnerable farm animal productions, such as freshwater fishing, aquaculture or grazing ruminants, and appropriate measures should mitigate the body burden of food-producing animals. Whereas the toxic nature of e-waste materials’ components is known, further cocktails of highly toxic (and lipophilic) chemicals are generated by bad practices such as open burning [34]. Improper practices (e.g., mechanical treatment and leaching) to recycle or dismantle the e-waste piles severely contaminate the environment (and, therefore, food chains) and operators [35,36]. Environmental disasters involve people occupationally exposed to and people living in proximity of polluted areas but also the population at large. Environmental contamination by these highly toxic chemicals is especially urgent now that the nutritional transition moves people from traditional diets (high in cereal and fiber) to diets high in fat and animal-source food. The deprived life environment of infants and children makes these life stages multifactorially and chronically exposed to severely toxic mixtures at higher levels than adults (more food, water and air per body weight). Often without adequate protection, child labor in highly severely toxic environments (e.g., e-waste but also mining and gas flaring) aggravates the scenario of acute and chronic high exposure. These vulnerable and susceptible situations overwhelm local capacity and should be recognized as an emergency injuring large numbers of people and demanding immediate international responsibility and action [5]. When international scientific literature allows it, also in data-poor contexts, the scientific evidence available worldwide allows context-specific alerts to risk managers on a severe health risk problem calling for action [34]. A diagnostic (or rapid) risk assessment based on real-life scenarios can demonstrate the existence of a problem and, eventually, estimate its magnitude and causes. Even in the absence of complete environmental and human internal exposure data, the environmental fate of toxicants is known and predictable and environmental pollution can be understood immediately [5].

The use of natural antidotes and probiotics with no side effects is being reconsidered for the treatment of human poisoning [37,38,39].

### 3.2. Knowledge Return Initiatives

Knowledge return initiatives empower communities against toxicological risk factors focused on (i) science-derived narration of hazards and exposure risks, (ii) information for healthy behaviors and informed choice on both consumer products and processes and (iii) science-based good practices to avoid or mitigate risk factors and protect health. When feasible and affordable, tools for awareness and empowerment in real-life exposure scenarios [40] included (a) media appearances (TV, radio and newspapers), (b) public talks and debates (e.g., town meetings), (c) participation in science cafés, (d) use of social media platforms and (e) involvement in collaborative research projects.

#### 3.2.1. Kitchen Toxicology

##### Educational Sessions on Breastfeeding and Baby Bottle Selection and Usage

**Figure 17:**
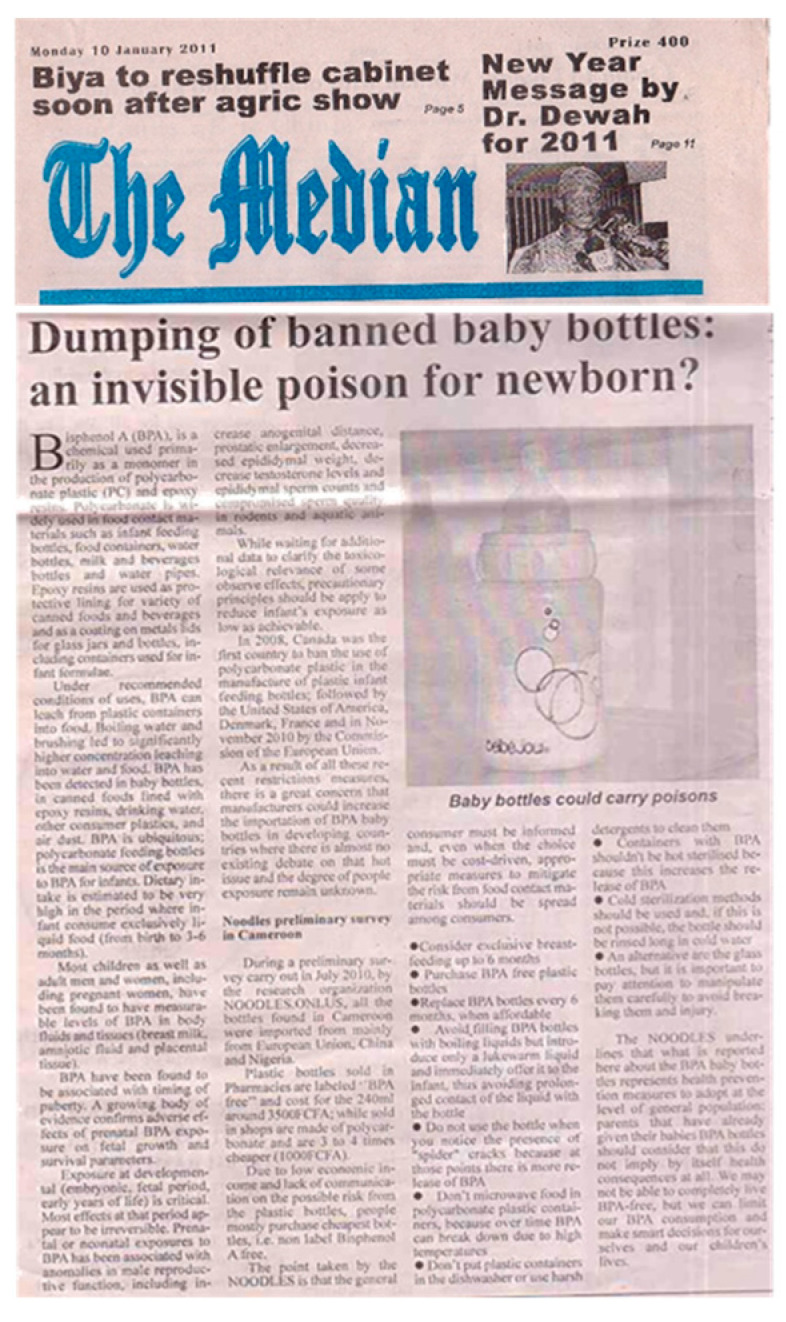
Informed choice and good practices on the safe use of Bisphenol A (BPA) baby bottles, The Median newspaper, Cameroon, 2011.

Interviews at vending sites and group discussions with mothers in local communities in Cameroon (counseling points and primary health care centers) focused on the harmful effects of BPA in baby bottles, informed choice and good practice [16]. The group discussions showed (i) the absolute absence of information and risk perception about toxic exposure via materials in contact with food, (ii) unawareness about the importance of materials and integrity of surfaces in contact with foods, (iii) unawareness of the meanings of products’ labels (when available) as well as iv) a high level of interest to gather information about labels, products and products’ usage patterns to avoid noxious exposure [16]. Early and exclusive breastfeeding is a highly recommendable health-promoting practice (for instance, breast milk is a source of iodine that is essential for the function of the thyroid and, hence, to body growth and intellectual development) that needs further support where, e.g., feeding water still is a common practice and neonates are not regularly fed colostrum. Breastfeeding avoids the risk of contaminated water. Bottle-feeding is increasingly widespread in urban settings to facilitate the nutrition of newborns whose mothers are employed. In the absence of top-down regulation and control of consumer products when daily real life makes product price a driver for consumer choices [41,42], consumers’ informed choice is central, as well as good practices avoiding everyday exposures (e.g., avoid filling boiling liquids and prolonged contact of the liquid with the bottle; replace after 6 months of use or in the presence of “spider” cracks; do not microwave, hot sterilize or put in a dishwasher; clean with soft detergents). Even in the case of illiterate consumers, labels with symbols are important in risk communication/health education. When health workers and vendors were interviewed in Cameroon and Nigeria, they showed no knowledge about dangerous toxic releases from plastic materials in foods under certain circumstances as well as the meaning of product labels indicating the presence/absence of toxic substances in baby bottles [16]. Good practices are needed to avoid toxic exposure from food contact materials, and feasible dissemination via radio programs as well as newspapers is confirmed (Figure 17).

##### Food Safety Education for Local Women to Avoid Toxicant Intake

Some recommendations were given to women planning pregnancy in Cameroon and Senegal [9,16]. Since they cannot significantly decrease their body burden because excretion of toxicants may require many years (most toxicants are persistent, i.e., with slow excretion/metabolic rates—e.g., dioxins, Polychlorinated biphenyls (PCBs) and Dichloro-Diphenyl-Trichloroethane (DDT), women at a childbearing age may avoid new intake of toxicants through proper selection of foods and a diet rich in green vegetables, fruits and eggs and low in fats and sugars. For instance, fish is a nutritious food, and factors such as fish age, species and size may significantly improve the benefits-to-risk ratio [24]. In general, recommendations such as consumption of lower-fat meats, fish, poultry and dairy products as well as removal of adipose tissues (e.g., skin and fatty portions) and bioaccumulating organs (e.g., liver, kidney) in foods of animal origin may effectively mitigate exposure to fat-soluble toxicants. Vegetables should be extensively washed to remove insecticides, pesticides and natural nitrates and clean water should be used to cook (often, water is reused for cooking after cleaning of equipment, utensils and raw foods). Based on the local diet, targeted assessment could increase the uptake of protective dietary factors, such as folates, antioxidants and essential trace elements (e.g., selenium and iodine), through dietary recommendations and indigenous foods. Noticeably, weight loss during pregnancy is discouraged as it increases fetus’ toxic exposure.

##### Good Practice Using Pesticides

Other good practices deserve main attention, such as those related to indoor residual spraying with DDT and pyrethroids; while spraying effectively controls malaria, it potentially increases human exposure, with an impact on neurodevelopment [43]. Illustrated practical guides allow the empowerment of both literate and illiterate people in urban, semi-urban and rural settings (e.g., [44]), whereas social marketing campaigns (e.g., [45]) could be adopted as well to reinforce the social change. For sure, health risk analysis and food safety from farm to consumption require ecological and anthropological approaches to clarify sources and dynamics of human health, diseases, diets, lifestyles and behaviors [9].

##### Food Nutrition Education at School

**Figure 18:**
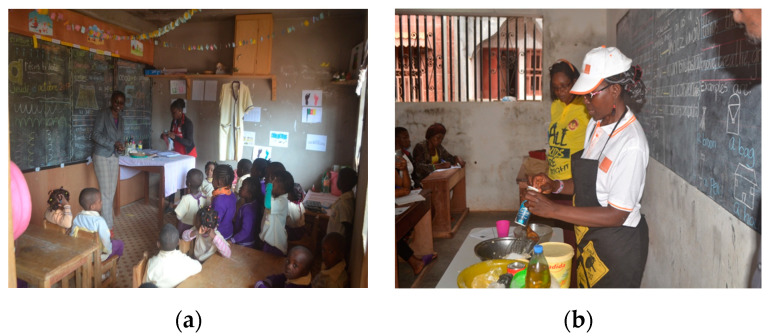
(**a**) Demonstrations to primary school children, St. Therese School, Cameroon, 2018; (**b**) Demonstrations to families and caregivers, Bright mind School and Tebap School, Yaoundé, Cameroon, 2018. “Primary schools project” (www.noodlesonlus.org) © courtesy of Noodles.

**Figure 19:**
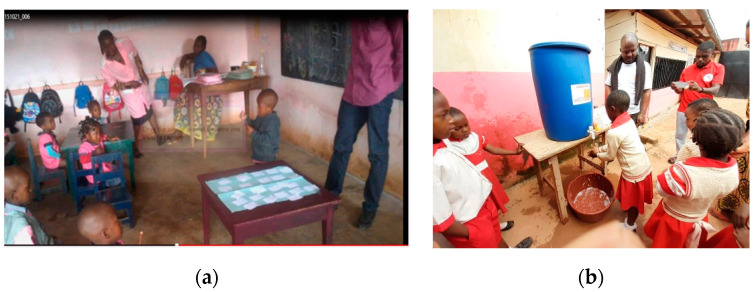
(**a**) Games on food groups, Cameroon, 2018; (**b**) Hand washing, St. Therese School and Omega School, Yaoundé, Cameroon, 2018. “Primary schools project” (www.noodlesonlus.org) © courtesy of Noodles.

The availability and accessibility of food alone cannot guarantee good nutrition, and food security programs should be leveraged to include healthy eating habits and encourage and enable safe and nutritious diets. Nutrition education in school curricula would benefit the promotion of adequate food, hygiene and sanitation practices as well as preventive and curative health. Despite advancements in international knowledge and scientific research on nutrition and food safety, the translation into awareness of consumers, citizens, school canteen workers and school administrators remains very poor. Since poor nutritional practices and toxic exposures can aggravate malnutrition [2], demonstrations of kitchen toxicology were offered to caregivers of primary school children. Such demonstrations covered basic good practices during handling, storing and transport of food to contribute seminal awareness towards protective measures against preventable noxious exposure (Figure 18). Education in primary schools promoted health-seeking behaviors and encouraged the school system to provide safer and nutritionally balanced meals, hygiene (starting from hand washing) and physical activity. As already proved by Mbhatsani et al. [46], basic notions on balanced nutrition (e.g., mainly based on fruit and vegetables, pulses, nuts, seeds, fish and seafood) through representation of food groups using colors, a food pyramid/food guide, relationships between nutrients, nutritional need and their functions and food classification by taste (Figure 19) were effective in imparting knowledge to primary school children as well improving dietary diversification with indigenous foods. As powerful agents of change, school children can be promoters of positive attitudes and healthy eating behaviors. In many African countries, the whole family eats the same meal from a common pot. “Special” recipes for young children, pregnant and/or breastfeeding mothers are not envisaged within these eating cultures. Engaging parents in healthy eating and food preparation and consumption reinforced the effectiveness of school-based safe nutrition education programs [45], whereas engaging the whole school system, including school managers/administrators and canteen operators, can support the sustainability of these effects. Food hygiene and safety education at the school level (hand washing, water sanitation and hygiene, safe cooking practices, including supply and storage, organization of appropriate kitchens and waste systems and organization of cost-effective school feeding programs including good practices to minimize food waste) through correct perceptions of the effects of food on health can support balanced nutrition of the community. This will also motivate shielding from initiatives promoting unsafe diets (e.g., junk snacks, i.e., those high in salt, sugar and saturated fats, and sugary drinks should be kept out of schools). Experiences of growth monitoring (anthropometric measurements, malnutrition scores and temperament) by teachers [47,48] demonstrate how toxic exposure indicators and risk factors for malnutrition and non-communicable diseases can be monitored early (e.g., obesity, dyslipidemia, hypertension, abnormal development, neurocognitive development, asthma, allergies and state of hair and nails). Last but not least, high primary school enrolment rates make the school a reference point (e.g., in Cameroon) in a systemic “nutrition and health” education that is essential for the diffusion of correct and updated information for consumers’ attitude towards informed choices and good practices for a healthy diet. Effective enrolment in public (often free) schools is expected to cover orphanages and also street children.

#### 3.2.2. Environmental Toxicology

##### Plastic and e-Waste Pollution

**Figure 20:**
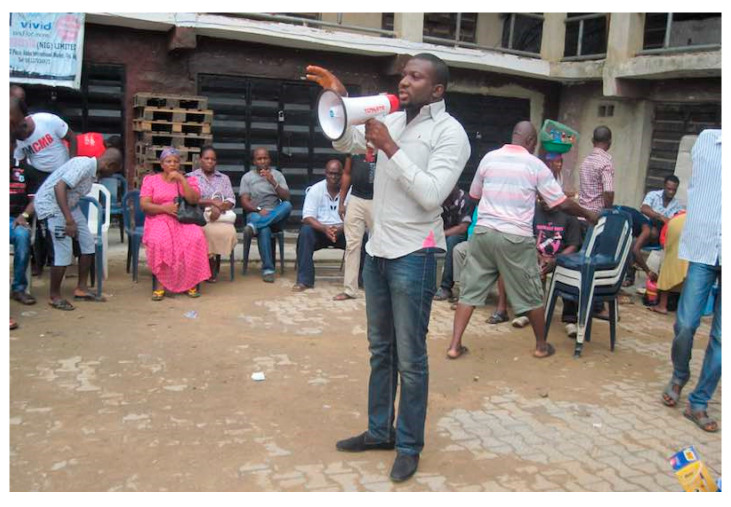
Occupational Health Hazards of E-Wastes: An Awareness Campaign at the Alaba International Market, Lagos, Nigeria, 2017 © Noodles (www.noodlesonlus.org).

Plastics may not only be a problem when they are used in contact with food but also when they are abandoned in the environment or dumped and improperly recycled. Electronic waste, or e-waste, contains both plastics (i.e., brominated flame retardants, phthalates, etc.) and metal components (i.e., toxic chemical elements); e-waste is dumped along river and costal banks where sometimes only a few usable modern ICT pieces can be salvaged. Bottom-up initiatives raised awareness at the Alaba International Market, Lagos State, Nigeria (Figure 20), to protect the locals from both occupational and environmental exposure to e-waste. Although some people claimed to have prior knowledge of the problem, further discussion clarified that a lot is yet to be known about occupational hazards and ways to avoid or minimize exposures, including personal protective equipment and adequate ventilation against fumes. Protective measures should include the families of e-waste workers, e.g., exposed clothes should be kept separate when being washed at home. The African scientific community has started studying solutions to the e-waste problem and to urge governments for a proper e-waste management system. Public health professionals are not sufficiently aware of the severe health consequences of e-waste exposure for the general population. Increased awareness and capacity building is crucial within the health sector. Awareness of the web of interactions and interdependence among environment–animals–humans (i.e., One Health) is the best tool to balance progress towards true development. Man-made contaminations impact ecosystems’ health, and the ecosystems make the contamination of food-producing animals and water a source of daily and repeated toxic exposure for the general human population. With the concept in mind, laboratory analyses of well water, feed and silage, individual feathers, fur, urines, and fecal samples for biomonitoring of live food-producing animals were collected by the local NGO to assess the feasibility of sentinel animal population for One Health [49,50].

##### Good Practice Education in Primary Food Production

With regards to food production, semi-intensive cultivation and rearing may introduce new and multiple health risk factors for humans, animals and the environment at large [26]. Networking and consultative groups of farmers and meat chain operators at divisional, sub-divisional and village levels were encouraged to adopt good practices via the NGO. Health risks in both short- and long-term of chemicals (e.g., biocides, fertilizers, pesticides, feeds’ ingredients and additives, veterinary drugs and antiparasitic drugs) intended for use in agriculture and zootechny were explained to agriculturists and agronomists with preliminary efforts employing easy and understandable toxicological charts. More comprehensive involvement and empowerment of the agro-farming and livestock systems have been overlooked until now, resulting in limited potential of local agro-zootechny in supporting safe nutritional security and counteracting the effects of climate changes [51]. It is known how maintaining crop diversity (e.g., underused food resources, such as sorghum [52]) can better preserve soil fertility, reduce the vulnerability to pests and diseases and improve nutrition.

## 4. Discussion

### 4.1. Information to Users and Consumers is One of the Keys to Avoid Toxic Exposure

Toxic exposure is often more a matter of awareness than affordability. Awareness of people, healthy informed choices and good practices and behaviors are risk management milestones towards disease prevention and health promotion; indeed, materials (also natural products) are chemicals and can affect health. Photos may disclose and facilitate narration of case risk scenarios in different cultural environments and boost preventive actions. With globalization, rapid changes in culture (e.g., lifestyle, diet and food production) constitute a non-negligible source of toxic hazards [4]. Food safety and environmental health concerns are growing in the African continent due to the increasing number of food crises and intoxications informed by mass media and the growing demand for quality products by African consumers [7].

### 4.2. Empowerment with Local Culture and Real-Life Scenarios

Some African countries have embraced CODEX Alimentarius Standards but surveillance is still not done effectively or not done at all due to generally weak or non-existent food safety systems [53]. Regulatory authorities’ inspections are partial and do not cover important hazards that require laboratory analysis [28]. In such unprotected scenarios, bottom-up proactiveness from research scientists can lower avoidable risks and bridge gaps between existing knowledge and the implementation of relevant findings [54]. The key to promote prevention of toxic exposure in Africa may lie in human relationships and cultural values. As already proven by widespread mutual help village associations, collectivism is highly valued in African societies. According to Léopold Sédar Senghor, a former President of Senegal, “In African society, technical activities are always linked with cultural and religious activities, with art and magic, if not with the realm of the mystical” (1976). Poor skills are an avoidable consequence of low national income, i.e., countries may achieve more with less, as seen in the Indian experience. The “awareness raising—community empowerment” vortex based on science-based (e.g., photographic) reportages may be valuable in promoting good practices against avoidable risk factors at the community (and larger) level in different contexts.

When fitting to needs, values and culture, empowerment fed with local real-life scenarios (subtending needs, values, culture and beliefs) and context-specific knowledge is expected:

(1) To stimulate science-based risks perception daily experienced by people;
(2) To facilitate communities in incorporating new knowledge, awareness of hazards and confidence to change harmful food habits and adopt positive healthy practices;
(3) To motivate effective behavioral change, new skills and preventive health-seeking behaviors;
(4) To encourage multidisciplinary clustering and discussion around topics of community health relevance;
(5) To allow maximizing the benefits from the use of a product while avoiding or at least minimizing risks (to human health or the environment, including animals) [55];
(6) To exploit community engagement towards a culture of prevention of health risk factors in both production and consumption of healthy products and environmental health;
(7) To stimulate scientific research in food and environmental toxicology in Africa, which is an increasing reality well worthy of attention [56];
(8) To solicit and motivate effective intervention and responsive policies in primordial and primary prevention.

### 4.3. Scientific Researches and Disseminations to the Local Communities

Science can promote dialogues between scientists and society [57] while new inputs acquired by proactive volunteers in the field may catalyze scientific research for the field of public health. The African scenario of fast-changing toxicological risks calls for combined science-based, bottom-up and top-down actions [54]. As found in the experiences of an African NGO Noodles, such configuration creates self-reinforcing feedback loops that provide the basis for adjustment to changing circumstances. Scientists will work within their own community as trusted community members in a vortex that—in the long term—will require engagement with the whole community [12]. Most of this is achievable even in the absence of much top-down input if acting and responding at the local level are encouraged and supported. The studies of anthropology and ecology equipped with illustration cards, photos and documentaries in native languages can facilitate context-effective analyses of health risk factors and culture-specific prevention as well as empowerment [9]. The understanding of community dynamics, social relations, costumes, habits, tacit rules and ways to circulate information is crucial. For instance, the social network and way of life may make “word of mouth” a very efficient mode of circulating information at the community level. Accessibility of science encourages conversation between people, professionals, scientists, local authorities and policy makers, thus facilitating formal top-down changes. Scientists can invest their knowledge in communication while health promotion for behavior changes will translate research findings into the policymaking process. The “vortex approach” can feed the motivation of local researchers in their role in the prevention system. Based on shared instructions, well-described methods and approaches for community participation, citizen science, which has already proved to be a successful partnership between volunteers and scientists to answer real problems [58,59,60], may be useful to collect *evidenza*. When a clear message promoting healthy choices and behaviors is given, African communities are ready to respond. Aspiration to next generation health does motivate a change of behavior.

### 4.4. Other Types of Toxicant Contaminations

Exempla given here are not exhaustive but exemplify how risk-to-benefit analyses of products and practices introduced in Africa with the global market are feasible despite resource constraints. Other instances of new products which are progressively substituting the artisanal ones include e.g., clothes made from artificial fibers treated with chemical dyes and flame retardants, phthalate-containing Polyvinyl Chloride (PVC) toys, medical devices, detergents and cosmetics and perfluorinated chemial-containing stain-resistant carpets. African markets deserve shielding from unsafe or hazardous foreign products while accepting products with high safety levels for both consumers and the environment. On the other side, higher awareness of toxicological risks is needed in local production of herbal medicines, drugs and cosmetics (e.g., red clays).

### 4.5. Primordial Prevention

While primary prevention aims at managing specific and individual risk factors and improving protective factors to reduce the incidence of diseases, primordial (or primal) prevention aims at establishing and maintaining conditions that prevent such risk factors, e.g., education, water and sanitation, housing, safe work environments and ecosystems. The severe problem of lead (Pb) exposure is well known, particularly in fast developing countries, such as Nigeria, caused by hazardous crude oil exploitation, mining, e-waste and exhaust emissions of vehicles. Field surveys proved how environmental exposure is exacerbated by other non-essential uses of Pb, such as Pb in paints in houses and schools, plumbing and solder pipes/tanks for drinking/household water storage, unsafe recycled Pb containing waste, including Pb acid batteries, and toys. Resources should, therefore, address both primary prevention and primordial prevention.

### 4.6. Power of Information Dissemination in Africa

Data exist but remain too sparse, unknown or underused in the decision-making process. In other cases, the information is altogether neglected. Field reportages focused on kitchen and environmental toxicology in daily life in the sub-Saharan African milieu can be used for risk analysis and awareness generation at all levels and communicate feasible recommendations for the empowerment of people. While food revolution increases local productions and recovers the great variety of vegetables and animal foods of the rich African food culture, global food trade requires the setting and implementation of safety standards [61]. Both clean food chains (i.e., with sustainable use of fertilizers, pesticides, veterinary drugs, sanitizers, etc.; good hygiene practices and measures to protect environmental safety) and a re-established value chain (supported by, e.g., control, certification and traceability) may provide young African enterprises an impetus for new business opportunities. Finally, when products pose significant health or environmental risks, ethical trade should enact bans on their use and export to any country [5]. When scientific data for risk assessment are either burdened by uncertainties or “insufficiency”, precaution should be regarded as a form of solidarity and contribution to development [62].

## 5. Conclusions

While African populations increasingly aspire to improve life expectancy in health, sub-Saharan Africa needs top-down choices for disease prevention as well as public awareness and attitudes towards health-protective choices and habits to face toxic exposures. Top-down actions alone, though energetic and good-willed, are unlikely to sufficiently enforce a culture praxis of safety and prevention in a society undergoing such fast and unregulated growth; the bottom-up support of motivated community energies can be of paramount significance in the process.

Tremendous efforts in promoting kitchen and environmental toxicology in Africa, a nowadays public health hot issue recognized in Africa, should be made. The contribution by an increasing number of universities is necessary for the engagement of a higher number of local research scientists working on the prevention of toxicological risk factors. Exposure to toxicants affects the health of people via multiple and various pathways and aggregated effects. An increasing collection of *evidenza* (or, when feasible, formal risk assessments based on evidence) will allow communities to define their specific risk scenarios, prioritize their risks, properly address available resources and, ultimately, make their science-informed choice of how to approach globalization and modernity.

## References

[1] Yankuzo K.I. (2013). Impact of Globalization on the Traditional African Cultures. J. Educ. Soc. Res..

[2] Frazzoli C., Mantovani A. (2020). Toxicological risk factors in the burden of malnutrition: The case of nutrition (and risk) transition in sub-Saharan Africa. Food Chem. Toxicol..

[3] Frazzoli C., Petrini C., Mantovani A. (2009). Sustainable development and next generation’s health: A long-term perspective about the consequences of today’s activities for food safety. Ann. dell’Istituto Super. Sanità.

[4] Frazzoli C., Mantovani A., Esposito R. (2016). Sustainable food safety and toxicant zoonoses: New prevention challenges in global health governance. Quad. della Soc. Ital. Med. Trop. Salut. Glob..

[5] Frazzoli C. The vulnerable and the susceptible: The weight of *evidenza* to stop exploiting activities generating toxic exposures in unprotected, data poor and deprived countries. J. Glob. Health.

[6] Ki-Zerbo J. (2011). Punti fermi sull’Africa. Antropolis.

[7] Mahajan V. (2009). Africa Rising: How 900 Million African Consumers Offer More Than You Think.

[8] Berrino F. (2010). Prevention and TAO. Epidemiol. Prev..

[9] Frazzoli C. (2020). Field anthropological research for context-effective risk analysis science in traditional cultures: The case of Senegal. J. Glob. Health Rep..

[10] Frazzoli C., Mantovani A. (2018). Editorial: The environment-animal-human web: A “One Health” view of toxicological risk analysis. Front. Public Health.

[11] Denning S. (2005). A Leader’s Guide to Storytelling: Mastering the Art and Discipline of Business Narrative.

[12] Sturmberg J.P., Njoroge A. (2017). People-centred health systems, a bottom-up approach: Where theory meets empery. J. Eval. Clin. Pract..

[13] Frazzoli C., Pouokam G.B., Mantovani A., Orisakwe O.E. (2016). Health risks from lost awareness of cultural behaviours rooted in traditional medicine: An insight in geophagy and mineral intake. Sci. Total Environ..

[14] Orisakwe O.E., Udowelle N.A., Azuonwu O., Nkeiruka I.Z., Nkereuwem U.A., Frazzoli C. (2019). Cadmium and lead in geophagic clay consumed in Southern Nigeria: Health risk from such traditional nutraceutical. Environ. Geochem. Health.

[15] Orisakwe O.E., Igweze Z.N., Ekhator O.C., Nwaogazie I., Frazzoli C. (2019). Appropriateness of essentials trace metals in commonly consumed infant formulae in Nigeria. Open Access Maced. J. Med Sci..

[16] Pouokam G.B., Ajaezi G.C., Mantovani A., Orisakwe O.E., Frazzoli C. (2014). Use of Bisphenol A-containing baby bottles in Cameroon and Nigeria and possible risk management and mitigation measures: Community as milestone for prevention. Sci. Total Environ..

[17] Niba L.K., Abia W.A. (2019). Waste-to-Opportunities: A Sustainable Option for Self-Reliance in the Informal Sector in Cameroon. MERC Glob. Intern. J. Manag..

[18] Wright C.Y., Godfrey L., Armiento G., Haywood L.K., Inglesi-Lotz R., Lyne K., Schwerdtle P.N. (2019). Circular economy and environmental health in low- and middle-income countries. Glob. Health.

[19] Kora A.J. (2019). Leaves as dining plates, food wraps and food packing material: Importance of renewable resources in Indian culture. Bull. Nat. Res. Cent..

[20] Orisakwe O.E., Frazzoli C., Frazzoli C., Asongalem E.A., Orisakwe O.E. (2012). Solid waste and waste management issues in sub Saharan Africa: A focus on Nigeria. Cameroon-Nigeria-Italy Scientific Cooperation: Veterinary Public Health and Sustainable Food Safety to Promote “One Health/One Prevention”.

[21] Frazzoli C., Mazzanti F., Achu M.B., Pouokam G.B., Fokou E. (2017). Elements of kitchen toxicology to exploit the value of traditional (African) recipes in the diet of HIV+/AIDS subjects: The case of Egusi okra meal. Toxicol. Rep..

[22] Proietti I., Frazzoli C., Mantovani A. (2014). Identification and management of toxicological hazards of street foods in developing countries. Food Cheml. Toxicol..

[23] Ekhator O.C., Udowelle N.A., Igbiri S., Asomugha R.N., Frazzoli C., Orisakwe O.E. (2018). Street foods exacerbate effects of the environmental burden of polycyclic aromatic hydrocarbons (PAHs) in Nigeria. Environ. Sci. Pollut. Res..

[24] Mantovani A., Ferrari D., Frazzoli C. (2015). Sustainability, security and safety in the feed-to-fish chain: Focus on toxic contamination. Internat. J. Nutr. Food Sci..

[25] Proietti I., Hoang D.V., Mantovani A., Frazzoli C. (2013). Protecting Vietnamese street food. New Agriculturist. Global Forum on Agricultural Research. http://www.new-ag.info/en/research/innovationItem.php?a=2984.

[26] Frazzoli C., Mantovani A. (2010). Toxicants exposures as novel zoonoses: Reflections on sustainable development, food safety and veterinary public health. Zoonoses Public Health.

[27] Cheng R., Mantovani A., Frazzoli C. (2017). Analysis of food safety and security challenges in emerging African food producing areas through a One Health lens: The dairy chains in Mali. J. Food Prot..

[28] Pouokam G.B., Foudjo B.U., Samuel C., Yamgai P.F., Silapeux A.K., Sando J.T., Atonde G.F., Frazzoli C. (2017). Contaminants in Foods of Animal Origin in Cameroon: A One Health Vision for Risk Management “from Farm to Fork”. Front. Public Health.

[29] Mantovani A., Frazzoli C. (2010). Risk assessment of toxic contaminants in animal feed. CAB Rev. Persp. Agric. Vet. Sci. Nutr. Nat. Res..

[30] Mantovani A., Frazzoli C., Cubadda F. (2010). Organic forms of trace elements as feed additives: Assessment of benefits and risks for farm animals and consumers. Pure Appl. Chem..

[31] Mantovani A., Frazzoli C., La Rocca C. (2009). Risk assessment of endocrine-active compounds in feeds. Vet. J..

[32] Frazzoli C., Gherardi P., Saxena N., Belluzzi G., Mantovani A. (2017). The hotspot for (global) one health in primary food production: Aflatoxin M1 in dairy products. Front. Public health.

[33] Ladeira C., Frazzoli C., Orisakwe O.E. (2017). Engaging One Health for non-communicable diseases in Africa: Perspective for mycotoxins. Front. Public Health.

[34] Frazzoli C., Orisakwe O.E., Dragone R., Mantovani A. (2010). Diagnostic health risk assessment of e-waste on the general population in developing countries’ scenarios. Environ. Impact Assess. Rev..

[35] Orisakwe O.E., Frazzoli C., Ilo C.E., Oritsemuelebi B.A. (2019). The public health burden of e-waste in Africa. J. Health Pollut..

[36] Frazzoli C., Mantovani A., Orisakwe O.E. (2013). Electronic Waste and Human Health. Reference Module in Earth Systems and Environmental Sciences.

[37] Amadi C.N., Offor S.J., Frazzoli C., Orisakwe O.E. (2009). Natural antidotes and management of metal toxicity in developing nations. Environ. Sci. Pollut. Res. Int..

[38] Amadi C.N., Frazzoli C., Orisakwe O.E. (2020). Nigerian foods of probiotic potentials and relevance to chronic metal exposure: A systematic review. Environmental science and pollution research. Environ. Sci. Pollut. Res..

[39] Orisakwe O.E., Nwadiuto Amadi C., Frazzoli C. (2020). Management of Iron overload in resource poor nations: A systematic review of phlebotomy and natural chelators. J. Toxicol..

[40] Ndlovu H., Joubert M., Boshoff N. (2016). Public science communication in Africa: Views and practices of academics at the National University of Science and Technology in Zimbabwe. J. Sci. Commun..

[41] Brimblecombe J., Ferguson M., Chatfield M.D., Liberato S.C., Gunther A., Ball K., Moodie M., Miles E., Magnus A. (2017). Effect of a price discount and consumer education strategy on food and beverage purchases in remote Indigenous Australia: A stepped-wedge randomised controlled trial. Lancet Public Health.

[42] Capewell S., Lloyd-Williams F. (2017). Promotion of healthy food and beverage purchases: Are subsidies and consumer education sufficient?. Lancet Public Health.

[43] Eskenazi B., Sookee A., Rauch S.A., Coker E.S., Maphula A., Obida M., Kogut K.R., Bornman R., Chevrier J. (2018). Prenatal exposure to DDT and pyrethroids for malaria control and child neurodevelopment: The VHEMBE cohort, South Africa. Environ. Health Perspect..

[44] Werner D., Thuman C., Maxwell J. (2020). Where There Is No Doctor. Edited by Hesperian health guides. https://store.hesperian.org/prod/Where_There_Is_No_Doctor.html.

[45] Blitstein J.L., Cates S.C., Hersey J., Montgomery D., Shelley M., Hradek C., Kosa K., Bell L., Long V., Williams P.A. (2016). Adding a social marketing campaign to a school-based nutrition education program improves children’s dietary intake: A quasi-experimental study. J. Acad. Nutr. Diet..

[46] Mbhatsani V.H., Mbhenyane X.G., Mabapa S.N. (2017). Development and Implementation of nutrition education on dietary diversification for primary school children. Ecol. Food Nutr..

[47] Mandiwana T.C., Mbhenyane X.G., Mushaphi L.F., Mabapa N.S. (2015). Knowledge and practices of pre-school teachers on growth monitoring program--South Africa. Health Promot. Int..

[48] Liu J., Xu X., Wu K., Piao Z., Huang J., Guo Y., Li W., Zhang Y., Chen A., Huo X. (2011). Association between lead exposure from electronic waste recycling and child temperament alterations. Neurotoxicology.

[49] Amadi N.C., Frazzoli C., Orisakwe O.E. Sentinel species for biomonitoring and biosurveillance of environmental heavy metals in Nigeria. J. Environ. Sci. Health Part C.

[50] Frazzoli C., Bocca B., Mantovani A. (2015). The one health perspective in trace elements biomonitoring. J. Toxicol. Environ. Health B Crit. Rev..

[51] Frazzoli C., Mantovani A., Dragone R. (2014). Local role of food producers’ communities for a Global One-Health framework: The experience of translational research in an Italian dairy chain. J. Agric. Chem. Environ..

[52] Proietti I., Frazzoli C., Mantovani A. (2015). Mantovani. Exploiting nutritional value of staple foods in world’s semi-arid areas: Risks and benefits, challenges and opportunities of sorghum. Healthcare.

[53] Pouokam G.B., Hamed H., Ngwafor R., Frazzoli C. (2016). Toxicovigilance systems and practices in Africa. Toxics.

[54] Frazzoli C., Mantovani A. (2019). Combining a bottom-up movement: Endocrine disruptors and non-communicable diseases in Africa. Open Access Government.

[55] Resnik D.B., Elliott K.C. (2015). Bisphenol A and risk management ethics. Bioethics.

[56] Frazzoli C., Asongalem E.A., Orisakwe O.E. (2012). Cameroon-Nigeria-Italy Scientific Cooperation: Veterinary Public Health and Sustainable Food Safety to Promote “One Health/One Prevention”.

[57] Orisakwe O.E., Frazzoli C. (2010). Water Supply in Niger Delta of Nigeria: From Public Protests to Scientific Discourse.

[58] Den Broeder L., Uiters E., ten Have W., Wagemakers A., Schuit A.J. (2017). Community participation in Health Impact Assessment. A scoping review of the literature. Environ. Impact Assess. Rev..

[59] Den Broeder L., Devilee J., Van Oers H., Wagemakers A. (2016). Citizen Science for public health. Health Prom. Internat..

[60] Cohn J.P. (2008). Citizen science: Can volunteers do real research?. Bioscience.

[61] Ng C.A., von Goetz N. (2017). The Global Food System as a Transport Pathway for Hazardous Chemicals: The Missing Link between Emissions and Exposure. Environ. Health Perspect..

[62] Petrini C. (2007). Poverty, human development, environmental and health risks: The role of precaution and cautionary policies. Ann. Ist. Super. Sanità.

